# Public feedback on a proposed statewide virtual translational research community

**DOI:** 10.1017/cts.2019.417

**Published:** 2019-09-12

**Authors:** Milton (Mickey) Eder, Christi A. Patten, Tabetha A. Brockman, Deborah Hendricks, Miguel Valdez-Soto, Maria Zavala-Rocha, Miriam Amelang, Chung Wi, Brittny Major-Elechi, Joyce (Joy) E. Balls-Berry

**Affiliations:** 1 Department of Family Medicine and Community Health, University of Minnesota, Minneapolis, MN, USA; 2 Center and Translational Science Institute, Academic Health Center, University of Minnesota, Minneapolis, MN, USA; 3 Center for Clinical and Translational Sciences, Mayo Clinic, Rochester, MN, USA; 4 Department of Health Sciences Research, Mayo Clinic, Rochester, MN, USA; 5 Mayo Clinic College of Medicine and Science, Rochester, MN, USA

**Keywords:** Social media, community engagement, translational science, clinical trials, recruitment

## Abstract

**Introduction:**

Researchers have explored using the internet and social media to recruit participants to specific research projects. Less systematic work has been done to inform the engagement of large populations in virtual communities to advance clinical and translational science. We report on our first step to use social media to engage Minnesota residents by studying the willingness of participants to engage in a virtual (Facebook) community about the concepts of health and health-related research.

**Methods:**

Data were collected at the 2018 Minnesota State Fair using a cross-sectional, 46-item survey with assessment including sociodemographics and willingness to engage in a Facebook group for health-related research. Quantitative analysis included univariate, bivariate, and multivariate analyses. Content analysis was used to generate themes from open-ended survey responses.

**Results:**

Five hundred people completed the survey; after data cleaning, 418 participant responses informed this report. A majority were younger than age 50 (73%), female (66%), and married/partnered (54%). Overall, 46% of participants agreed/strongly agreed they are willing to join the Facebook group. Multivariate logistic regression identified social media use over the past 6 months as the sole variable independently associated with willingness to join the Facebook group (once a day vs. never or rarely OR = 1.82 (0.86, 3.88), several hours a day vs. never or rarely OR = 2.17 (1.17, 4.02, overall p-value 0.048).

**Conclusion:**

Facebook holds potential for reaching a broader community, democratizing access to and engagement with clinical and translational research. Social media infrastructure and content could be disseminated to other institutions with Clinical and Translational Science Awards.

## Introduction

In *We Need to Talk*, Celeste Headlee asks us to think about how to participate actively in conversation [1]. How do we know when conversations start and end? How do conversants decide what topics are appropriate? How do they take turns talking or transition to new topics? Are there different social cues and norms for face-to-face (synchronous) and internet (synchronous and asynchronous) conversations? How much of medium is the message? We, community-engaged researchers at the Mayo Clinic (CCaTS) and University of Minnesota (CTSI) NIH-funded Centers for Clinical and Translational Science, plan to explore the use of social media to engage Minnesotans in conversations about health and clinical research. In looking to augment traditional face-to-face strategies by engaging community members in conversations using social media, our first step was to seek public feedback on their willingness, readiness, and reservations about participating in a digital community focused on health and research.

We report on a study to learn about public interest in developing a social media (Facebook) community that will focus on health and research as primary topics of conversation [2]. Our long-term goal is to sustain an online community of diverse voices and perspectives from across Minnesota. We not only expect social media to democratize access to information about health and research, we also expect social media to help transform how clinical research is designed and implemented with an eye toward optimizing how research produces benefit and improves community health. In seeking to reach and engage a diverse public, we further expect participants to coalesce into distinct online communities to converse on particular topics and identities.

Social media has the potential to extend the reach of translational science to diverse communities. The Pew Research Center reports that 89% of Americans use the internet with use and nonuse rates comparable across diverse racial and ethnic groups (i.e., Whites, Blacks, and Hispanics). Nonuse correlates with age, education, household, and community type with almost a third of seniors (= 65 +) and a third of Americans with less than high school education reporting no internet use. Twice as many rural residents are nonusers by comparison to urban and suburban residents [3,4]. Regarding adult users, Pew Research found 68% of internet users use Facebook, with a smaller proportion actively communicating through Instagram (35%) and Twitter (24%) [6]. Three-quarters of those 68% of adult Facebook users spend almost 1 hour per day on Facebook. Moreover, two-thirds of all Americans report that they get at least some news through social media [5]. Clearly, Americans are using social media and over time the social media user base has also grown more representative of the broader population [6].

While researchers have explored using the internet and social media to recruit participants for specific research projects, use of social media to engage diffuse populations to advance translational science remains largely untested. Here we share survey responses from Minnesotans at the State Fair or annual “Great Minnesota Get-Together” about their interest and willingness to engage in a Facebook group to have conversations on social media about health and research.

## Materials and Methods

Data for this cross-sectional survey study were collected in 2018 at the Minnesota State Fair Driven to Discover Research Facility; the facility serves as a venue to support fairgoer engagement with primarily minimal risk research projects. The largest in the Midwest, more than 2 million people attended the fair in 2018 [7] and over 60,000 fairgoers visited the Driven to Discover Research building [8]. While study staff stood outside the Driven to Discover building to inform those passing by of research opportunities, study staff inside talked with individuals who stopped by our research display *Like, Follow, Share*, explaining the project and inviting their participation in our “Like Follow Share: #MNResearch” study.

A sample size of 500 was deemed sufficient for determining public willingness to engage in a Facebook group. We anticipated conservatively that 50% of respondents would have a high Likert score for the primary outcome variable on the survey. To achieve a 5% margin of error, we needed 384 respondents to complete the survey. We estimate 20% (*n* = 76) of the surveys may be incomplete or blank. To account for this, we increased our sample size to 500 [9]. Enrolling 500 participants would support bivariate and multivariate analyses of demographic characteristics associated with willingness to engage, enabling further determination of small to medium effect size.

### Eligibility and Enrollment

Study staff within the Driven to Discover Research building provided detailed information about the study to individuals who expressed interest. Individuals were given time to ask questions and decide whether or not they wished to participate. Interested participants were screened by study staff to determine eligibility according to the following criteria: (a) resident of Minnesota, (b) 18 years of age or older, and (c) able to provide oral consent. Eligible participants who provided verbal consent were handed an iPad with an initial screen containing a link. Clicking on the link was interpreted as indicating voluntary informed consent; the link activated the survey. Study staff were readily available to assist individuals in the process of their taking the survey. Participants submitted their completed surveys by touching a button on the screen. After clicking on the submit button, a new link appeared with the question “Would you like to receive the findings from this study.” Those indicating “Yes” were instructed to provide an email address with an accompanying explanation that the survey they had already submitted would not be linked to this email address. All survey participants received a $20 Visa gift card as a thank you for their time.

Data were collected over 3 days during the fair and stopped only when the IRB approved number of participants was reached. A total of 500 people completed the survey of which 13 were removed from the analysis because their survey response indicated a zip code in another state. The sample was reduced by an additional 4 participants who did not answer the question about willingness to join a Facebook group and reduced again by eliminating the responses of 65 participants who indicated they had not created a personal Facebook profile. Thus, after data cleaning, the study consisted of 418 participants, all of whom had a Facebook profile. For the multivariate analyses, we clarify the criteria from the univariate analyses to enter the model for each variable’s association with willingness was *p* < 0.05. SAS 9.4 was used for data analyses.

This study was approved by the Mayo Clinic and the University of Minnesota Institutional Review Boards.

### Measures

The research team developed and piloted the survey to assess content and duration. The survey included 46 items administered electronically on an iPad using Qualtrics software. It required between 10 and 20 minutes to complete with the variation partly dependent on individual facility using the iPad technology. The items analyzed in this report included:

#### Sociodemographics

Sociodemographic variables included gender, biological sex, age group (18–29, 30–49, 50+), race, ethnicity, education level, employment status, marital status, zip code (used as proxies for rurality and median household income), health literacy [10], and prior participation in a health research study.

**Fig. 1.**
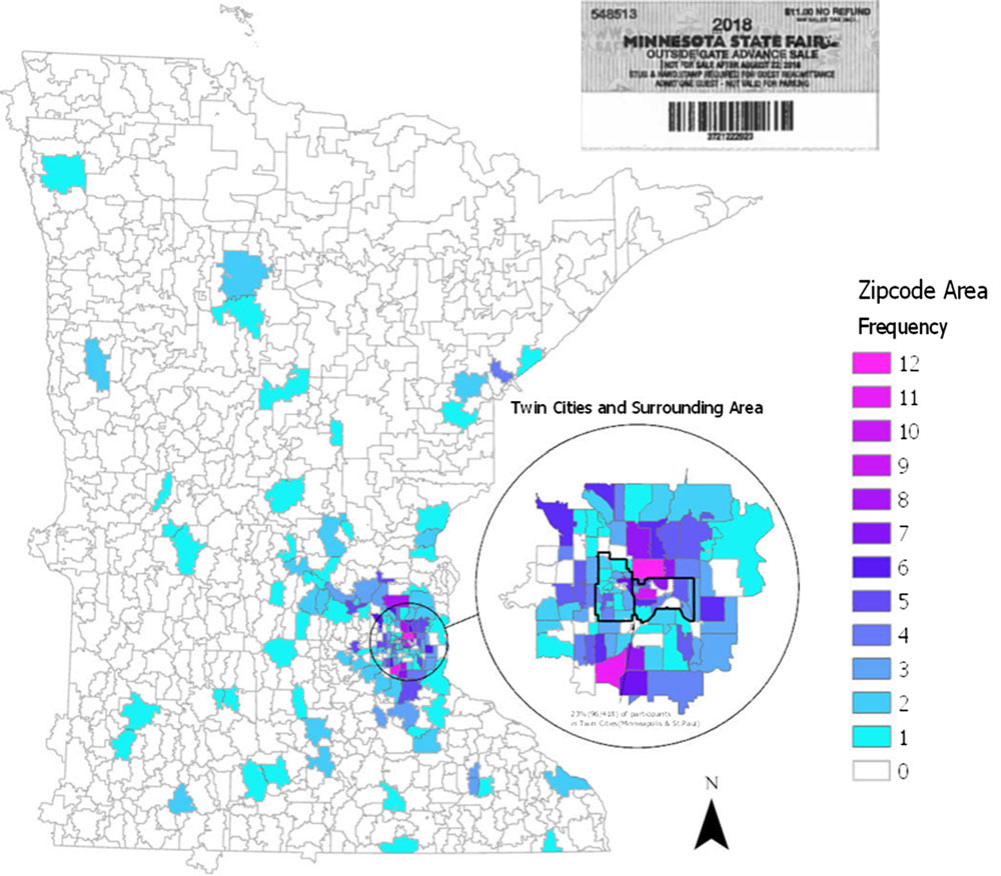
Geographical distribution of survey participants by zipcode.

Zip code data allowed us to determine the rurality of participants according to the Rural-Urban Commuting Area (RUCA) codes. While RUCA codes were developed by the US Department of Agriculture to capture tract-level rurality, the Center for Rural Health has provided a ZIP code approximation of the 2010 RUCA codes [11] For this study, we define rural as all nonmetropolitan zip code areas (RUCA1–3 = Urban) or Rural (RUCA = 4–10) [12]. Zip code data allowed us to map where survey participants reported living in Minnesota (Fig. 1) and to use the 2015 American Community Survey to estimate median household income as a surrogate marker for participants’ socioeconomic status

#### Social Media Use

Participants marked the social media platforms for which they had created a personal profile (e.g., Facebook, Instagram, LinkedIn, WhatsApp, Snapchat, twitter, YouTube, and Other). In addition, participants were asked to report how often in the past 6 months they had interacted with social media using a four-point scale ranging from never to several times a day.

#### Attitudes Toward Health Research

Participants were asked twice to assess their feelings about health research using a five-point Likert scale from strongly disagree to strongly agree: (1) “I feel health research can benefit my health and/ the health of others (e.g., family, community)” and (2) “There are some things about health research that I do not trust at all.” An open-ended response requested participant feedback: “Please give examples of your trust in health research.”

#### Willingness to Join a Facebook Group for Health Research (Dependent Variable)

Participants were asked “What do you think about joining a Facebook group to learn about health research in Minnesota?” and prompted “I would be willing to be part of this Facebook group,” with response options on a Likert scale: strongly disagree, disagree, undecided, agree, or strongly agree.

#### Facebook Group Preference Types – Information, Health Topics, and Engagement Strategies

Three open-ended responses were used to obtain feedback on motivating participation in a Facebook group: “What type of information would you like to get through this Facebook group?”, ”What health topics would you want to learn about as part of this Facebook group?”, and “What should we include in this Facebook group to keep people engaged and interested?”

### Data Analysis

#### Quantitative Analyses

Descriptive statistics using frequencies and means were used to summarize participant characteristics including sociodemographics, social media use, and mistrust in health research (Table 1).

**Table 1.** Minnesota survey participant sociodemographics, social media use, and attitudes toward health research (N = 418)

Characteristic	% (*n*)^a^
Age in years	
18–29	37.2 (155)
30–49	35.5 (148)
50+	27.3 (114)
Biological sex	
Male	34.0 (142)
Female	66.0 (276)
Gender^b^	
Male	34.0 (142)
Female	65.3 (273)
Transgender	0.2 (1)
Genderless	0.5 (2)
Race	
White	78.7 (326)
African American	6.3 (26)
Asian	4.8 (20)
American Indian	1.0 (4)
Multiracial	6.5 (27)
Other	2.7 (11)
Hispanic ethnicity	7.0 (29)
Education	
Less than high school	0.5 (2)
High school degree/GED	10.0 (42)
Some college	21.8 (91)
Associate degree	6.0 (25)
College degree	32.5 (136)
Graduate degree	29.2 (122)
Residence status^c^	
Rural (RUCA code >= 4)	10.0 (42)
Urban (RUCA code 1–3)	90.0 (376)
Estimated median household income^d^	
Low ($24,688–$60,805)	33.7 (140)
Medium ($61,340–$74,264)	34.0 (141)
High ($74,342–$120,718)	32.3 (134)
Employment	
Student	16.5 (69)
Not employed	4.8 (20)
Retired	7.2 (30)
Employed full- or part-time	71.5 (298)
Marital status	
Single (includes divorced/widowed)	45.9 (192)
Married/partnered	54.1 (226)
Health literacy: need assistance to read materials from doctor/pharmacy	
Never	68.7 (287)
Rarely	16.7 (70)
Sometimes	10.3 (43)
Often	2.6 (11)
Always	1.7 (7)
Interacted with social media in past 6 months	
Never	2.7 (11)
Rarely	11.6 (48)
Once a day	15.7 (65)
Several times/day	70.0 (289)
Created personal profile on social media platform	
Instagram	61.2 (256)
Snapchat	50.5 (211)
Twitter	45.7 (191)
LinkedIn	47.1 (197)
YouTube	37.8 (158)
Whatsapp	20.1 (84)
Ever participated in a health research study	32.8 (138)
“I feel research can benefit my health and/or others (e.g., family, community)”	
Strongly disagree	1.7 (7)
Disagree	0.2 (1)
Undecided	5.3 (22)
Agree	36.2 (151)
Strongly agree	56.6 (236)
“There are some things about health research that I do not trust at all”	
Strongly disagree	12.2 (51)
Disagree	25.4 (106)
Undecided	32.8 (137)
Agree	22.2 (93)
Strongly agree	7.4 (31)

a Percentages are based on non-missing data. Some percentages do not add to 100 due to rounding.

b Gender endorsement was 98.8 % concordant with biological sex endorsement

c Rural-Urban Commuting Area Codes (RUCAs) based on zip code data.

d Estimated income based on zip code data. Categories are based on sample distribution of estimated income.

The *χ*-square test was used to compare willingness to join the Facebook group by sociodemographic characteristics and social media use (Table 2). Multivariate logistic regression was used to assess variables in Table 2 that were independently associated with willingness to join the Facebook group. Variables that were significant at the bivariate level were included in the multivariate model (Table 3). Significance was set *a prior* at 0.05. For these analyses, participants who agreed/strongly agreed were compared to a group combining all other responses.

**Table 2.** Associations of participant sociodemographics and willingness to be part of a facebook group for biomedical research (N = 418)

	*Willing to be* *part of the* *facebook group*^a^		
Characteristic	*Yes* *N = 191*	*No* *n = 227*	*p-value*^b^
Age in years			0.299
18–29 (*n* = 155)	45.8 (71)	54.2 (84)	
30–49 (*n* = 148)	50.0 (74)	50.0 (74)	
50+ (*n* = 114)	40.4 (46)	59.6 (68)	
Biological sex			0.040
Male (*n* = 142)	38.7 (55)	61.3 (87)	
Female (*n* = 276)	49.3 (136)	50.7 (140)	
Gender			0.038
Male (*n* = 142)	38.7 (55)	61.3 (87)	
Female (*n* = 272)	49.5 (135)	50.5 (138)	
Race			0.26
White (*n* = 326)	145 (44.5)	181 (55.5)	
Racial minority (*n* = 88)	45 (51.1)	43 (48.9)	
Hispanic ethnicity			0.917
Yes (*n* = 29)	44.8 (13)	55.2 (16)	
No (*n* = 384)	45.8 (176)	54.2 (208)	
Education			0.398
High school /GED or less (*n* = 44) Some	34.1 (15)	65.9 (29)	
College/Associates (*n* = 116)	48.3 (56)	51.7 (60)	
College degree (*n* = 136)	47.8 (65)	52.2 (71)	
Graduate degree (*n* = 122)	45.1 (55)	54.9 (67)	
Residence status			0.090
Rural (*n* = 42)	33.3 (14)	66.7 (28)	
Urban (*n* = 376)	47.1 (177)	52.9 (199)	
Estimated median household income			0.328
Low (*n* = 140)	50.7 (71)	49.3 (69)	
Medium (*n* = 141)	45.4 (64)	54.6 (77)	
High (*n* = 134)	41.8 (56)	58.2 (78)	
Employment			0.912
Not employed/student/retired (*n* = 119)	45.4 (54)	54.6 (65)	
Employed full- or part-time (*n* = 298)	46.0 (137)	54.0 (161)	
Marital status			0.885
Single/divorced/widowed (*n* = 192)	45.3 (87)	54.7 (105)	
Married/partnered (*n* = 226)	46.0 (104)	54.0 (122)	
Interacted with social media past 6 months			0.014
Never or rarely (*n* = 59)	28.8 (17)	71.2 (42)	
Once a day (*n* = 65)	44.6 (29)	55.4 (36)	
Several times a day (289)	44.6 (29)	50.5 (146)	
Health literacy: need assistance to read materials from doctor/pharmacy			0.034
Never (*n* = 287)	43.9 (126)	56.1 (161)	
Rarely (*n* = 70)	40.0 (28)	60.0 (42)	
Sometimes/often/always (*n* = 61)	60.7 (37)	39.3 (24)	
Ever participated in a health research study			0.086
Yes (*n* = 135)	51.9 (70)	48.1 (65)	
No (*n* = 282)	42.9 (121)	57.1 (161)	

Values are % (*n*). Percentages are based on non-missing data.

a Willingness defined as endorsing “agreed” or “strongly agreed” to the question “I would be willing to be part of this Facebook group.”

b *χ*-square test.

**Table 3.** Multivariate logistic regression on the factors associated with willingness to participate

Variable	Level	Odds ratio (95 % CI)	*p*-value
Sex	Female	1.52 (0.99–2.33)	0.053
Health literacy	Never	Reference	0.09^a^
	Rarely	0.86 (0.5–1.48)	0.59
	Sometimes/often/never	1.81 (1.01–3.22)	0.045
Social media use	Never or rarely	Reference	0.048^a^
	Once a day	1.82 (0.86–3.88)	0.12
	Several hours a day	2.17 (1.17–4.02)	0.01

a overall *p*-value.

#### Qualitative Analyses

Content analysis was used to generate prominent themes from responses to open-ended questions [13]. Two authors independently (TAB and MZR) coded and demonstrated a high level of inter-rater reliability (κ = 0.859, 95% CI, *p* < .0005) [14]. Different interpretations were discussed and resolved with input from a third author (CAP).

## Results

### Participants

Of the 418 participants, 73% were under 50 years of age, 21% indicated a race other than White, 66% identified as female, and 54% were married or partnered (Table 1). Ten percent of the sample resided in a rural area based on zip code, which is less than the calculated 27% of the rural population of Minnesota [15], or the 15%–19% rural residents across the US population. About one-third of participants lived in zip code areas with a low household income status ($24,688–$60,805) in a state with a 2016 median income of $63,217.

About 15% of participants acknowledged needing some assistance with reading materials from a doctor or pharmacy. Of note, the proportion expressing health literacy challenges was related to age (*p* = 0.015) and higher among those aged 50 and older (43.1%) as compared to either those aged 19–29 (19.3%) or 30–49 (12.8%). Health literacy challenges did not differ by sex.

In considering the past 6 months, most participants (70%) interacted with social media several times per day (Table 1). In addition to having a personal profile on Facebook, participants also reported personal profiles on Instagram (61%), Snapchat (50%), LinkedIn (47%), and Twitter (46%).

### Attitudes Toward Health Research

While one-third of the sample had previously participated in a health research study, 93% of participants agreed or strongly agreed that research can benefit their own health or the health of others (family/community). However, 30% of participants agreed/strongly agreed that there are some things about health research that they do not trust.

Participants (*n* = 250) commented on mistrust in health according to the following themes:

1. *Funding Sources* (i.e., bias and conflicts of interest) “Any study designed by Big Pharma, most recently the ‘opiates are safe for chronic pain studies,’” “Biases from companies,” “Depends what the sources of funding are,” and “Funding sources drive results;”
2. *Racial biases in research “Any results specific to gender and race. Are there biases in research that I am not aware,” “Does not benefit everyone,”* and *“Equity and diversity of research subjects;”*
3. Bias related to small sample sizes “Limited studies and small sample sizes,” “size of the sample group,” “Oftentimes the research group includes more inner city and not rural folks like me,” and “diversity of research subjects;”
4. Different and conflicting research findings/reports “Conflicting results from different studies,” conflicting research can make things confusing, and hard to believe future research,” and “knowing who to believe with so many opinions;”
5. Accuracy, credibility, and reliability of results and information in research reports “Funding source drives results,” “I like to know if the results are reproducible,” “It can be difficult to determine credibility of online sources,” “Knowing if it is a reliable resource or not,” and “Lack of peer review;”
6. Confidentiality “Data mining and confidentiality,” “Government access to info,” and “Can be used against you;”
7. *Privacy* “*Online privacy,” “Privacy of medical records*.”

### Willingness to Join a Facebook Group for Health Research

Almost half (46%) of participants reported being willing to join (agree/strongly agree) this Facebook group to learn about health research in Minnesota. Bivariate associations between willingness to join a Facebook group for biomedical research and sociodemographic characteristics support the following claims. Willingness to join the Facebook group was significantly associated with participant sex (females: 49% vs. males 39%; *p* = 0.040) and gender (*p* = 0.038), with social media interactions in the past 6 months (several times a day (45%), once a day (45%), never/rarely (29%); *p* = 0.014) and with health literacy (sometimes/often/always needing assistance (61%), never (44%) and rarely (40%); *p* = 0.034) (Table 2).

Multivariate logistic regression identified social media use over the past 6 months as the sole variable independently associated with willingness to join the Facebook group (once a day vs. never or rarely OR = 1.82 (0.86, 3.88), several hours a day vs. never or rarely OR = 2.17 (1.17, 4.02, overall *p*-value 0.048) (Table 3)**.**

### Facebook Group for Health Research: Information, Health Topics, and Engagement Strategy Preferences

Table 4 presents content analysis sorted themes and illustrative quotes for the types of information, health topics, and engagement strategies that participants thought should be included in a Facebook group for health research. As the Table illustrates, participants wanted information about participating in research, about new research findings, health and wellness, health care access and resources. Participants wanted guidance to credible and reliable health information. Health topics of interest included health and wellness, chronic disease prevention (e.g., cancer), mental health, aging, and infectious and autoimmune disorders. Recommended ways to engage and keep people interested in the Facebook group included attention grabbing content that utilizes the multimedia potential of social media (e.g., short stories, videos, trivia games, or polls). Participants reiterated concern for the quality of information.

**Table 4.** Preferences for facebook group information, health topics, and ways to keep people engaged: Themes and illustrative quotes from content analysis^a^

Themes	Illustrative quotes
What types of information would you like to get through this Facebook group? (*n* = 203)	
1. Participating in research and new research findings	“How to participate in health research studies” “Invites to participate in research projects” “Availability of different research studies, research findings” “Any new result of health research current and upcoming research” “New breakthroughs in research”
2. Health and wellness	“General health issues” “Healthy living” “Health promotion” “Fitness, weight loss, circadian rhythm, recovery from physical stress” “Health issues to address things that affect Minnesotans for which they can do something about (pragmatic) such as sun screen, repellents, food prep, healthy eating, etc.” “Innovations in healthcare”
3. Health care access and resources	“Actions to take if an illness arises” “Health care information” “Different clinics around me” “How to be healthy and get best care possible for reasonable price, explore health insurance issues and how to make cost of health care more affordable for everyone”
4. Credible and reliable resources for health information	“A qualified and expert voice to confirm or deny health related issues and new fads” “Academic articles” “Links to research articles directly from the journals “researchers” were published in” “Links to legitimate sites” “Reliable and valid health information”
What health topics would you want to learn about as part of this Facebook group? (*n* = 213)	
1. Health and wellness	“How to better take care of yourself and healthier eating habits” “Healthy lifestyle choices” “Physical activity,” “Exercise research” “Nutrition” “Weight loss,” “Obesity” “General wellness”
2. Chronic disease and prevention	“Cancer”, “Cancer treatment,” “Cancer research,” “Cancer topics” “Heart health,” “Heart issues,” “Heart disease prevention, “Tachycardia” “Respiratory,” “Asthma” “Diabetes” “Disease prevention” “Preventative care, chronic illness maintenance” “Living with chronic disorders”
3. Mental health	“Mental health awareness or any other types of health related things that people don’t think about in their day to day lives, even though it can impact them in their day to day lives like having a healthy diet and getting enough exercise” “Mental health” “Anxiety,” “Depression” “ADHD”
4. Aging	“Alzheimer’s and other conditions of older people” “Joint health, preventive activities of diseases that come along with aging” “Healthy aging, women’s health” “Elder care” “Senior health” “Health topics for people over 50”
5. Infectious and autoimmune disorders	“Things that are local or Midwest. Tick borne diseases, water-related” “Lyme’s disease” “HIV/AIDS” “Infectious diseases, travel med” “Different research on topics of common illnesses (cold, flu, etc.)”
What should we include in this Facebook group to keep people engaged and interested? (*n* = 218)	
1. Attention grabbing content	“2 min run-downs. People won’t read full articles” “Visuals that catch the eye when scrolling” “Images, infographics…something to catch the eye in my newsfeed” “Neil deGrasse Tyson state of mind when presenting science to the public it should be fun informative and something that directly affects people’s lives” “Informative videos and relevant stories” “Videos explaining everything being done” “A balance of pictures and text” “Be straight to the point without cluttering up the feed” “Fun facts that are short but interesting…something that does not take a long time to read” “Easy ability to jump to topic of interest”
2. Include new research findings	“Always updating with new topics. Keep it interesting and change it frequently to keep your audience” “Frequent Posts with clever attention ‘grabbers’” “Make it user friendly, avoid complicated medical terms so people of all ages and social classes may understand” “Real stories not just facts” “Personal stories and meaningful statistics ““Short stories with from people with disease”
3. Interactive activities	“Current research links” “Links to articles” “Recent advancements, treatments, outreach” “Promising new drug findings and treatments” “New topics and articles explaining new research” “Easy to read research documentation” “Gear it for the average lay person” “Lots of explanations of previous research, and why this kind of research is important”
4. Credible and reliable information	“Health related Trivia” “Interactive dialogues, video content, quizzes or assessments or other exercise that keep people interacted” “Discussion boards” “Surveys, community engagement, opportunities to share opinions” “Daily questions” “Maybe there could be like a check in type thing or survey or something where people would actually have to participate in a questionnaire or something. Gotta keep it relevant and interactive for the people” “Poll could be an interesting way to keep individuals engaged” “Rewards/prizes help engage most people (like raffles or the gift card for this survey)”, “Free giveaways” “Keep it extremely professional so you can build some clout and report” “Facts relevant to the research” “Focus on solid research and facts, avoid ‘baiting” headlines (these diminish credibility” “Quality articles, no click bait” “Real data and research methods”

a Inter-rater agreement on themes for two independent raters (TB, MZ) was excellent (Cohen’s *kappa* coefficient = 0.86). Discrepancies in coding were discussed with a third author (CP) until agreement was reached.

## Discussion

“Like Follow Share: #MNResearch” survey responses indicate that almost half of Minnesotans who are already interacting through Facebook social media profiles are willing to participate in a Facebook group around health and research. Participants expressed interest in access to credible health information and in using social media to address health literacy issues or challenges they face in decoding health information. Participants tempered their interest in research by sharing concerns about bias in research and conflicts of interest, both issues of scientific integrity. Mistrust in relation to research is a consistent recurring issue [16]. However, almost half the survey participants indicate support for development of a social media platform to explore health and research.

Science cafés and community engagement studios [17–19] exemplifies successful face-to-face engagement strategies. Some more unique face-to-face programs like HealthStreet and Boot Camp Translation [20,21] include a personal component that is not easily disseminated. While face-to-face engagement programs offer a solid foundation on which to build a social media platform, limitations include accessing local events and scale. We want to explore whether social media offers a scalable and sustainable engagement practice.

Developing a Facebook group holds the possibility of reaching a broad public and democratizing access to and engagement with clinical and health services research. The Facebook social media platform also makes possible support for members of subgroups to share particular interests or discuss personal experiences related to health conditions and/or research participation. The ability to adjust criterion for group membership and limit sharing through privacy settings further expands the potential value of refining virtual communities.

Formative work at Mayo Clinic indicates that social media can be used to engage underrepresented, diverse community members in time-limited, two-way dialogs about health research [22,23]. Individuals who participated in face-to-face Garden Café forums indicated they desired to continue dialogs about research on social media venues (Facebook, blog, Twitter) [18]. Evaluation of these early efforts supports the utility of developing a social media platform for ongoing, two-way communication and co-learning across diverse populations.

Social media can foster coordination of community engagement in clinical and biomedical research activities across various academic-community committees (e.g., Cancer Centers, Prevention Research Centers, community advisory boards, practice-based research networks, public health organizations, and patient and community advocacy groups) that may help address community concerns about information credibility and reliability and research biases. Sharing information provides opportunities to increase transparency and foster trust. The relative ease of spreading information broadly and repeated individual exposure to messages both inside and outside Facebook groups will allow us to explore and apply social media conversation cues and rules to translational science [11,24].

Social media also offers opportunities to engage patients and other stakeholders in raising awareness of clinical trials and to understand and address barriers to clinical trial participation in both urban and rural contexts [25–29]. It provides a platform for patients and community members to disseminate stories about their research experience. Additionally, social media dialogs that include community and patient voices and perspectives make available opportunities to shape oversight of research conduct for new areas of research such as broad consent initiatives, including individual management of content in health information databases and biospecimens in storage.

At least three questions require immediate attention: first, what resources are needed to generate content and manage a social media group? Second, are there strategies for sharing information and promoting bidirectional engagement in biomedical research that increases participant knowledge and shapes attitudes about the perceived value of clinical and translational research? And finally, how do we capture and study engagement and user-generated content to assess effectiveness of different digital communication modalities in improving health and increasing participation in research?

## Limitations

Our analytic approach was chosen to assist with practical application of our findings. We wanted to understand, in a preliminary fashion, the proportion of people in MN who might be interested in joining the FB group. We could then target certain groups in our outreach efforts who may be more or less interested. Although focusing on a dichotomous outcome with a stronger level of agreement (willing/not willing) with an item is a conservative approach, it seems reasonable that time and resources should be spent on developing a new social media platform for community engagement only if there is an overall reasonable proportion of our sample willing to join.

## Conclusion

Virtual communities constitute an emerging but untested approach to engaging communities in translational science. Feedback to guide development of an online community obtained through the *Like Follow Share* study indicated public concern about the credibility of information, variability of research findings, and a lack of trust regarding research conduct and oversight. However, public willingness to participate in an online community focused on health and research among adult Facebook users in Minnesota suggests that these concerns can be addressed through ongoing social media interaction. Demonstrating that social media can sustain engagement of a virtual community in conversations and active engagement in health and clinical research would constitute a platform worthy of dissemination to other institutions with Clinical and Translational Science Awards.
